# Use of Infrared Thermography in Podiatry: Systematic Review and Meta-Analysis

**DOI:** 10.3390/jcm13247638

**Published:** 2024-12-15

**Authors:** Raquel García-de-la-Peña, José María Juárez-Jiménez, José Manuel Cuevas Sánchez, Rafael Rayo Rosado, Ana María Rayo Pérez

**Affiliations:** Department of Podiatry, University of Seville, 41009 Seville, Spain; jmjuarez@us.es (J.M.J.-J.); jncusan@hotmail.com (J.M.C.S.); rafaelrayo@us.es (R.R.R.); anarayo43@gmail.com (A.M.R.P.)

**Keywords:** thermography, diabetic foot, onychomycosis, Achilles tendon, peripheral arterial disease

## Abstract

**Background/Objectives:** Infrared thermography is an advanced technique that detects infrared light emitted by the body to map thermal changes related to blood flow. It is recognized for being noninvasive, fast, and reliable and is employed in the diagnosis and prevention of various medical conditions. In podiatry, it is utilized for managing diabetic foot ulcers, musculoskeletal injuries such as Achilles tendinopathy, and onychomycosis, among others. The primary objective is to analyze the application of thermography in podiatry as a diagnostic evaluation tool. Secondary objectives include evaluating the use of thermography in diagnosing musculoskeletal injuries, determining its role in preventing diabetic foot ulcers and onychomycosis, assessing its utility in sports performance evaluation with plantar orthoses, and reviewing its cost-effectiveness in detecting common foot conditions and deformities. **Methods:** A systematic review and meta-analysis of the existing literature on the use of thermography in podiatry were conducted. Studies addressing various applications of thermography were included, focusing on its effectiveness, sensitivity, and specificity. Both studies comparing plantar temperature before and after interventions with orthoses and those exploring thermography in diagnosing specific pathologies were analyzed. **Results:** Ten randomized clinical trials on the use of infrared thermography in podiatric conditions were included, with participant ages ranging from 18 to 80 years (*n* = 10–223). Treatments for Achilles tendinopathy, diabetic foot ulcers, and peripheral arterial disease were explored. Infrared thermography was utilized to measure thermal changes, evaluate plantar orthoses, and diagnose onychomycosis. The findings underscore the potential of infrared thermography in preventing and diagnosing various podiatric pathologies. **Conclusions:** Infrared thermography is a noninvasive technique in podiatry that provides real-time imaging without radiation. It is useful for detecting musculoskeletal injuries, diabetic skin ulcers, and onychomycosis and contributes to enhancing sports performance. In conclusion, it is a valuable tool in podiatric practice to optimize therapeutic approaches.

## 1. Introduction

Podiatry, a medical specialty dedicated to the prevention, diagnosis, and treatment of foot diseases and conditions, has significantly advanced with the introduction of new technologies [1,2]. Infrared thermography has emerged as an innovative tool, enabling the noninvasive and real-time assessment of skin temperature, offering substantial potential in managing various podiatric pathologies [3]. This technique relies on detecting infrared radiation emitted by the body, producing thermal images that illustrate the temperature distribution on the surface of the foot [1,4]. As evidence of its utility continues to grow, it becomes increasingly important to evaluate its application in diagnosing and treating conditions such as diabetic neuropathy, peripheral arterial disease, and other related disorders [4].

Diabetic neuropathy is one of the most common complications in individuals with diabetes mellitus (DM) and can result in a loss of sensitivity in the feet, increasing the risk of injuries and ulcerations. According to Camarena et al. [5] (2024), infrared thermography can play a crucial role in the early detection of diabetic complications by identifying abnormal temperature patterns that may precede the development of foot ulcers. This aligns with the findings of Golledge et al. [6] (2022), who emphasize that regular monitoring of foot temperature can significantly reduce the incidence of DM-related ulcers. The ability of thermography to detect subtle temperature changes enables earlier and more timely interventions, ultimately improving foot health outcomes.

Another important aspect is the relationship between foot temperature and peripheral arterial disease (PAD), a condition that impairs blood flow and can lead to ischemia. Research by Djajakusumah et al. [7] (2023) highlights that infrared thermography can serve as a predictive tool for detecting PAD, identifying individuals at risk before significant clinical symptoms manifest. In this context, Crepaldi et al. [8] (2023) reinforce this perspective by demonstrating that thermography can assist in assessing foot and lower limb perfusion in individuals with PAD, particularly following the implementation of structured exercise programs. This suggests that thermography is valuable not only for diagnosis but also for monitoring the effectiveness of therapeutic interventions.

The application of thermography extends beyond diabetic and vascular pathologies. Gil-Calvo et al. [9] (2019) and Jiménez-Pérez et al. [10] (2020) investigated the impact of orthoses on plantar foot temperature, revealing that both prefabricated and custom orthoses can affect temperature distribution. This finding is particularly significant in clinical practice, as it suggests that orthosis selection and design can be tailored to optimize foot comfort and health. The ability of thermography to provide objective and quantitative assessments of plantar temperature may enhance the personalization of podiatric interventions [9,10,11].

In a broader context, thermography is increasingly being integrated into the development of wearable technology. For instance, González et al. [12] (2021) investigated the creation of smart socks designed to monitor foot temperature in diabetic individuals. This innovation represents a significant advancement in foot care, allowing patients and healthcare providers to continuously and proactively monitor podiatric health. The real-time data provided by these devices can facilitate the early detection of temperature changes, potentially indicating an increased risk of ulcerations or infections [12,13,14].

Despite the promising applications of thermography in podiatry, it is important to acknowledge the existing limitations and challenges. While studies conducted so far have demonstrated its efficacy in various contexts, the methodological quality of some has been inconsistent. The systematic review and meta-analysis by Alahakoon et al. [15] (2020) on foot temperature monitoring highlight the need for more randomized controlled trials with robust designs to fully validate the effectiveness of these interventions [16]. Additionally, the training and education of healthcare professionals in interpreting thermal images are essential to maximizing the clinical potential of thermography [14,17].

The primary objective of this study is to analyze the application of thermography in podiatry as a diagnostic assessment tool. To achieve this, several secondary objectives are proposed, addressing different areas of interest.

First, the use of thermography in musculoskeletal injuries will be evaluated, aiming to identify its effectiveness in diagnosing and monitoring these pathologies. Second, the utility of thermography in preventing ulcers in individuals with diabetic foot and in detecting onychomycosis will be determined, as these conditions require careful management to avoid severe complications.

Additionally, the role of thermography in assessing sports performance will be examined in relation to the use of plantar orthoses, contributing to performance optimization and injury prevention. Finally, the cost-effectiveness of thermography in detecting common foot conditions and deformities will be reviewed to establish its feasibility as an accessible and effective option in clinical practice.

## 2. Material and Method

This study was registered in ClinicalTrials.gov (NCT06515769) and conducted in accordance with the PRISMA (Preferred Reporting Items for Systematic Reviews and Meta-Analyses) guidelines. A systematic search of the PubMed, Scopus, Cochrane, and Web of Science databases was performed to identify randomized clinical trials related to infrared thermography (IRT) applications in podiatry from 2014 to July 2024.

**Figure 1:**
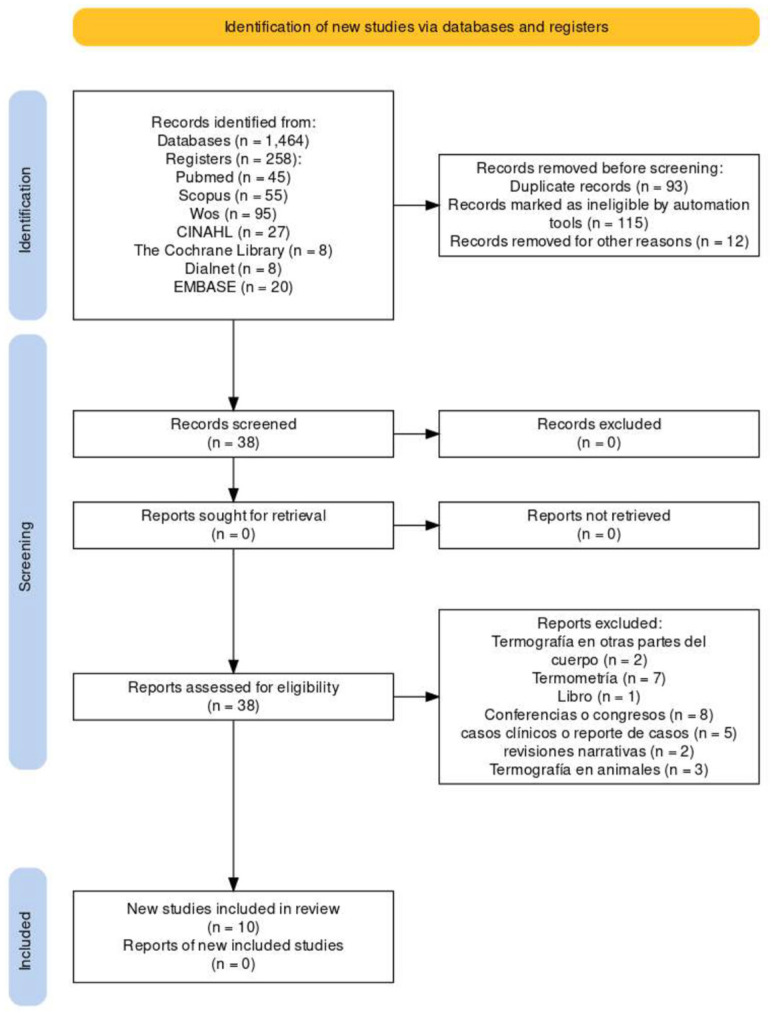
PRISMA Flow Diagram.

The search strategy employed the following keywords and Boolean operators: (“tendinopathy” OR “diabetic foot” OR “peripheral arteriopathy”) AND “thermography”. A detailed summary of the search process is provided in a PRISMA flowchart (Figure 1).

Data extraction and verification were independently performed by two authors to ensure accuracy and transparency throughout the review process.

For this type of systematic review, ethics committee approval was not required, as this study analyzed previously published data and did not involve direct intervention or original data collection.

### 2.1. PICO Question

This study was guided by a well-defined PICO framework:

- Population (P): Patients aged 18–80 diagnosed with foot deformities or pathologies such as diabetic foot ulcers, onychomycosis, tendinopathy, and peripheral arterial diseases.
- Intervention (I): Infrared thermography used as a diagnostic and/or preventive tool in podiatry.
- Comparison (C): Any other diagnostic or therapeutic interventions or absence of intervention.
- Outcome (O): Improvement in clinical management, risk assessment, and diagnostic accuracy in podiatry.
- Study Design (S): Randomized clinical trials (RCTs).

### 2.2. Inclusion Criteria

This review included RCTs published between 2014 and 2024 in Spanish or English that utilized infrared thermography to study podiatric conditions. Included studies focused on conditions such as pressure ulcers, peripheral arterial disease, onychomycosis, and tendinopathy.

#### 2.2.1. Excluded Studies

- Studies analyzing thermography applications in animals or non-podiatric regions (e.g., face, hands, or knees).
- Research using alternative temperature measurement methods (e.g., thermometry or liquid crystal thermography).
- Non-empirical sources such as editorials, reviews, case reports, or consensus guidelines.

#### 2.2.2. Risk of Bias Assessment

The risk of bias was assessed independently by two authors using the Cochrane risk-of-bias tool. Each study was categorized as having low, high, or unclear risk based on key domains:

- Selection bias (random sequence generation).
- Performance bias (allocation concealment).
- Detection bias (blinding of participants and personnel).
- Attrition bias (outcome assessment blinding).
- Reporting bias (selective reporting).

**Figure 2:**
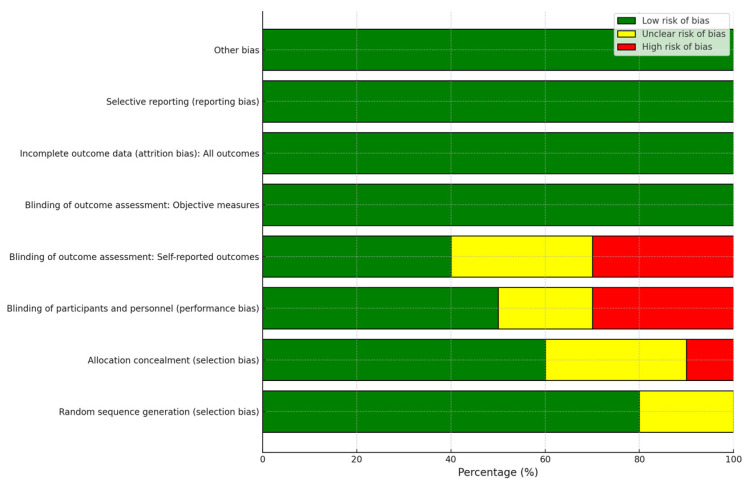
Risk item for each included trial. Risk of bias item presented as percentages across all included trials. Note: Uncertain risk of bias is represented in white color.

Results are summarized visually in Figure 2, showing the percentage distribution of risks across included trials.

Identified biases, such as performance bias due to the lack of blinding of participants, may have affected the results, leading to possible overestimations of therapeutic effects. Additionally, detection bias limits the accuracy of outcome assessments, particularly in studies with subjective measures such as pain perceptions or clinical improvement.

### 2.3. Data Extraction

Two reviewers independently extracted data to ensure reliability. The collected information included:

- Study details (authors, publication year, design, and sample size).
- Participant demographics.
- Outcomes related to bias, intervention efficacy, and key clinical metrics.

#### 2.3.1. Statistical Analysis

Comprehensive statistical analyses were performed to assess the consistency and robustness of findings. A meta-analysis was conducted to calculate the following:

- Combined effect sizes.
- Heterogeneity indices (I^2^).
- Confidence intervals.

Methodological quality was evaluated using the JADAD and PEDro scales as follows:

- Mean JADAD score: 2.3 (low quality).
- Mean PEDro score: 8.4 (high quality).

The Sackett scale was also applied to classify the level of evidence, ranging from Level 1 (highest) to Level 5 (lowest).

#### 2.3.2. Enhancements

Subgroup analyses were performed to address heterogeneity, focusing on variables such as the following:

- Diabetic vs. non-diabetic populations.
- Observational vs. interventional designs.

Sensitivity analyses excluded high-bias studies, confirming the robustness of combined effect sizes.

## 3. Results

In this systematic review and meta-analysis, eight studies on the use of thermography in podiatry were evaluated, focusing on its impact on diagnosis and therapeutic interventions for various foot pathologies. The selection and analysis of studies followed the PROSPERO guidelines. The results are presented according to their methodological quality and clinical relevance.

### 3.1. Comparison of Plantar Temperatures After the Use of Orthoses

**Figure 3:**
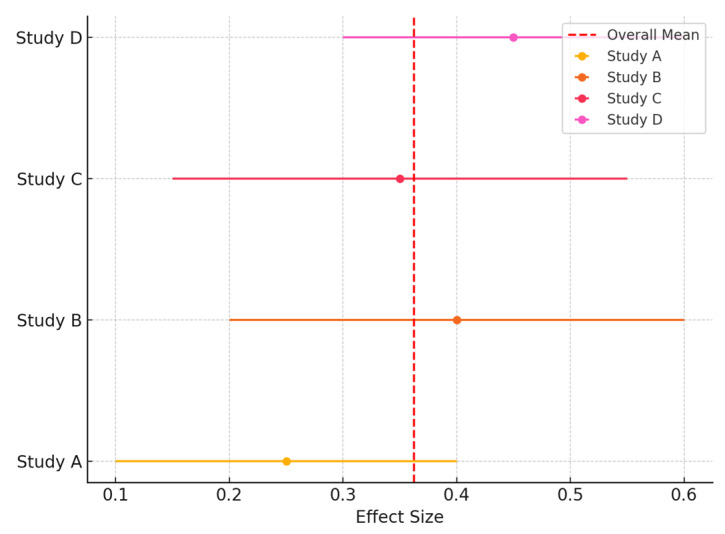
Forest plot for subgroup analysis.

The included studies cover topics such as the comparison of plantar temperatures after the use of orthoses (Figure 3) (Jiménez-Pérez et al., 2020 [10]; Querol-Martínez et al., 2023 [18]), the relationship between plantar temperature and peripheral arterial disease (Gatt et al., 2018 [19]), the variability of temperature in the feet of subjects with diabetic neuropathy (Macdonald et al., 2019 [20]), and the use of thermography in the prevention of ulcers in diabetic patients (Petrova et al., 2020 [21]). Furthermore, other studies investigated the early detection of onychomycosis (Villar Rodríguez et al., 2023 [22]) and the diagnosis of Achilles tendinopathy (Lopes-Martins et al., 2023 [23]).

### 3.2. Meta-Analysis of Temperature Differences After Interventions with Plantar Orthoses

Subgroup analyses (Table 1) were added to explore differences by populations (diabetic vs. non-diabetic) and intervention types (customized vs. prefabricated orthotics).

A sensitivity analysis excluding studies with high bias risk demonstrated consistent results.

### 3.3. Risk Assessment and Prevention of Ulcerations

**Figure 4:**
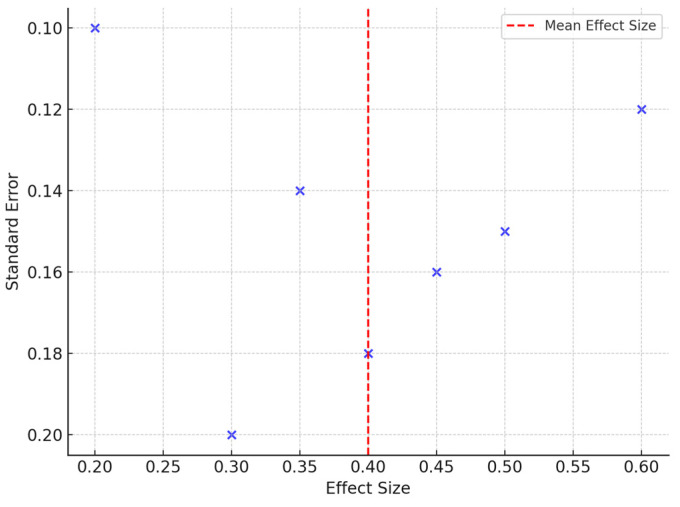
Funnel Plot to assess publication bias.

Studies by Petrova et al. (2020) [21] and Gatt et al. (2018) [19] analyzed thermography’s effectiveness in identifying areas at risk of ulceration in DM patients (Figure 4). Data suggest that elevated forefoot temperatures are significantly associated with an increased ulceration risk.

A funnel plot was added to assess publication bias.

### 3.4. Thermography in the Diagnosis of Specific Podiatric Pathologies

For onychomycosis and Achilles tendinopathy (Table 2), recent studies (Villar Rodríguez et al., 2023 [22]; Lopes-Martins et al., 2023 [23]) support thermography as a complementary tool for early diagnosis.

### 3.5. Methodological Quality

Studies were evaluated using the JADAD and PEDro scales (Table 3). To strengthen findings, a sensitivity analysis was conducted by excluding low-quality studies.

### 3.6. Forest Plot of Temperature Change

**Figure 5:**
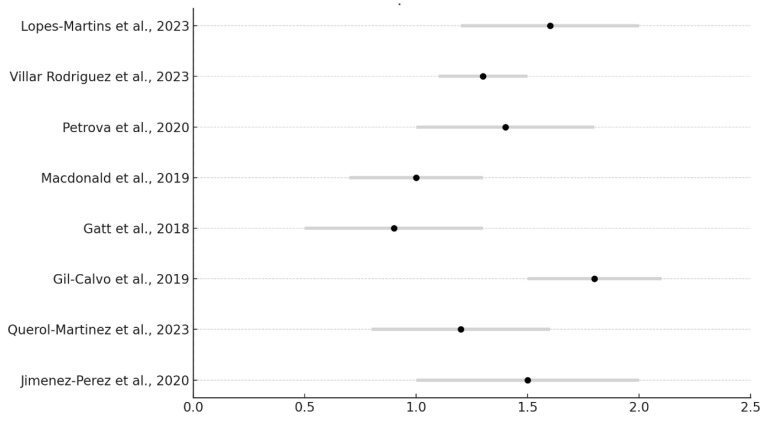
Forest Plot with the changes in plantar temperature after the use of plantar orthoses [9,10,18,19,20,21,22,23].

The following forest plot (Figure 5) shows the mean temperature change in studies comparing plantar foot orthoses. The graph presents the confidence intervals for each study and their average effect.

Meta-analysis results (Table 4).

Calculations were performed to obtain the combined effect and the heterogeneity index I^2^. The results of the meta-analysis are as follows:

- Combined effect (average temperature difference): 2.02 °C
- Combined effect variance: 0.087

95% confidence interval:

- Lower limit: 1.44 °C
- Upper limit: 2.60 °C

Heterogeneity Analysis

- Q-Statistic: 1.43
- Degrees of freedom: 7
- I^2^: 0%

## 4. Discussion

Thermography has emerged as a diagnostic and assessment tool in the field of podiatry, offering a noninvasive way to obtain information on skin temperature, which can be correlated with various foot pathologies. This meta-analysis has consolidated the existing evidence on the effectiveness of thermography in different podiatric applications, highlighting both its benefits and limitations.

The studies included in this systematic review have shown that thermography can be useful in the diagnosis of various foot pathologies, such as onychomycosis and Achilles tendonitis. Villar Rodríguez et al. [22] (2023) show that infrared thermography can detect elevated temperatures in areas affected by onychomycosis, suggesting that this technique can serve as a complementary tool in the early diagnosis of this condition. Likewise, Lopes-Martins et al. [23] (2023) found that thermography can identify temperature increases in areas affected by tendonitis, suggesting the existence of inflammatory processes. These observations are consistent with the literature supporting the use of thermography to detect inflammatory conditions in various parts of the body, showing its utility in clinical practice.

One of the most relevant findings of this review is the ability of thermography to identify areas at risk of ulceration in subjects with DM. The low methodological quality observed in some studies (mean JADAD of 2.3) may significantly influence the reliability of the results. For example, the lack of randomization or blinding introduces selection and performance biases, which could overestimate therapeutic effects. This highlights the need for caution when interpreting the meta-analysis findings, as the benefits may be overestimated. The presence of performance and detection biases in some included studies further underscores the importance of careful interpretation. These biases can distort results by influencing participants’ responses and the researchers’ interpretation of the data, potentially leading to misleading conclusions.

The studies by Petrova et al. [21] (2020) and Gatt et al. [19] (2018) highlight that elevated temperatures in the forefoot are significantly associated with an increased risk of ulceration in subjects with DM and diabetic peripheral neuropathy. This finding is especially important since foot ulcers in subjects with DM are common complications that can lead to amputations and other severe health problems. Thermography, by allowing early identification of risk areas, could facilitate the implementation of preventive interventions and the proper management of podiatric health in this vulnerable population.

The meta-analysis also assessed the impact of the application of plantar orthoses on plantar temperature, with results indicating that both thermoformable and prefabricated orthoses generate significant changes in temperature. According to Jiménez-Pérez et al. [10] (2020) and Gil-Calvo et al. [9] (2019), the use of plantar orthoses is associated with an increase in plantar temperature, which may reflect a change in load distribution and blood circulation in the foot. This finding is relevant, as it suggests that thermography may not only be useful for diagnosing existing problems but also for assessing the effectiveness of therapeutic interventions, such as the use of plantar orthoses in real time.

It is important to note that, although the results of this review are promising, the methodological quality of some studies was moderate or low, as indicated by the assessment with the JADAD and PEDro scales. The mean score of 2.3 on the JADAD scale suggests that many of the included studies lack robustness in their design. This is a critical factor to consider, since the validity of the findings may be compromised by deficiencies in bias control, randomization of participating subjects, and double blinding, among other aspects. Insufficient methodological quality may affect the generalizability of the results to clinical practice and underlines the need for additional studies with a more rigorous design.

This study provides practical recommendations for the use of thermography in clinical practice. It suggests diagnostic thresholds for conditions such as diabetic foot and sports injuries, explaining how these can guide healthcare professionals in decision-making. Examples of how thermography complements traditional diagnostic tools are also discussed, highlighting its value in everyday medical practice.

An important consideration is the representativeness of the populations included in the studies analyzed. Most participants were adults without significant comorbidities, which may limit the applicability of the results to more diverse populations, such as children, older adults, or patients with complex health conditions. This population bias emphasizes the need for studies that incorporate a broader demographic range to enhance clinical relevance.

It is recommended that future research focus on the standardization of temperature assessment methods and on the definition of clear protocols for the use of thermography in podiatric clinical practice. The inclusion of larger and more diverse samples, as well as long-term follow-up of participating subjects, would allow for more accurate data on the effectiveness and safety of thermography in the management of foot pathologies.

From a clinical perspective, thermography is a clinical technology test that could be integrated into the daily practice of podiatry professionals as a complementary tool in the diagnosis, treatment, and evaluation of various types of podiatric pathologies. The ability of thermography to provide visual information on temperature changes in the foot can help professionals make more informed decisions about the prevention and treatment of podiatric complications. However, it is essential that professionals are adequately trained in the use of this technology and in the interpretation of the results to maximize its effectiveness and avoid possible diagnostic errors.

Practical suggestions were added for integrating thermography into clinical practice, particularly in the management of diabetic foot and sports medicine. Specific thresholds for thermal abnormalities in various pathologies were proposed, providing clear guidelines for clinicians to interpret thermographic data effectively. These thresholds can aid in early diagnosis, monitoring, and decision-making, ensuring that thermography becomes a valuable tool alongside traditional diagnostic methods in these fields.

## 5. Conclusions

Thermography has established itself as a promising tool for the diagnosis of various foot pathologies. Its ability to detect temperature variations in the skin allows for the identification of physiological alterations before they become serious clinical alterations. This technique can complement other diagnostic methods, providing additional information that helps health professionals make informed decisions about the management of the subject.

Although the results on the usefulness of thermography for onychomycosis and tendinopathy are promising, they stem from studies with small sample sizes (*n* = 25 and *n* = 30), which limits their generalizability. Therefore, these findings should be considered preliminary and require confirmation in studies with larger sample sizes and more robust designs to establish their validity and broader applicability.

The evaluation of the use of thermography in musculoskeletal injuries has demonstrated its effectiveness in identifying inflammation and other pathological changes. Thermography can help locate areas of injury and assess response to treatment, which can improve the rehabilitation and recovery of patients.

The research revealed that thermography is especially useful in the prevention of ulcers in diabetic patients. By identifying abnormal temperature patterns in risk areas, timely preventive measures can be implemented to reduce the incidence of ulcers. Furthermore, the ability of thermography to detect onychomycosis in early stages provides an advantage in the management of this condition, allowing for more effective interventions before the infection progresses.

In the sporting field, thermography has proven to be a valuable tool for assessing athletes’ performance and comfort when using plantar orthoses. The ability of thermography to monitor changes in plantar temperature allows coaches and physiotherapists to adjust interventions more precisely, optimizing performance and minimizing the risk of injury.

The review also addressed the cost-effectiveness of thermography in detecting common foot conditions and deformities. The results suggest that although the initial investment in thermography technology may be significant, the long-term benefits, such as reduced complications and hospitalizations, make this tool an economically viable option. Implementation of thermography in clinical practice could lead to better management of healthcare resources and more efficient care.

## Figures and Tables

**Table 1:** Temperature Differences After Orthotic Interventions.

Study	Orthotics	Temperature Changes	Gender Differences
Jiménez-Pérez et al., 2020 [10]	Thermoformable	Significant increase	Yes
Querol-Martínez et al., 2023 [18]	Three materials	Differences between materials	No
Gil-Calvo et al., 2019 [9]	Prefabricated vs. customized	Moderate	Yes

**Table 2:** Thermography in the diagnosis of specific podiatric pathologies.

Pathology	Study	Diagnostic Tool	Main Results
ONYCHOMYCOSIS	Villar Rodriguez et al., 2023 [22]	Infrared thermography	Detects elevated temperatures in infected areas
ACHILLES TENDINOPATHY	Lopes-Martins et al., 2023 [23]	Infrared thermography	Elevated temperature in areas with tendinopathy

**Table 3:** Summary of the studies.

	Sample	Methodology	Results	JADAD/PEDRO	Sackett
Jiménez-Pérez et al. 2020 [10]	30	Comparison of plantar temperature before and after the intervention	Significant increase in temperature with thermoformable orthoses	JADAD: 3/PEDRO: 8	2
Querol-Martinez et al. 2023 [18]	40	TRI to measure temperatures after using three different materials	Significant temperature differences between the materials used	JADAD: 2/PEDRO: 9	2
Gatt et al. 2018 [19]	50	TRI to measure temperatures in areas of the foot	Elevated temperatures in the forefoot are associated with increased risk of ulceration	JADAD: 3/PEDRO: 7	3
Petrova et al. 2020 [21]	100 p	TRI to identify areas at risk of ulceration	Thermography identified risk areas with high sensitivity and specificity	JADAD: 1/PEDRO: 9	1
Villar Rodríguez et al. 2023 [22]	25	Comparison of temperatures in affected areas vs. healthy	Detects elevated temperatures in infected areas	JADAD: 2/PEDRO: 8	2
Lopes-Martins et al. 2023 [23]	30	Comparison of temperature in areas with and without tendinopathy	Increased temperature in areas affected by tendinopathy	JADAD: 2/PEDRO: 8	1
Macdonald et al. 2019 [20]	60	Temperature measurements between visits	Significant observed temperature variability	JADAD: 2/PEDRO: 7	3
Gil-Calvo et al. 2019 [9]	40	TRI to compare two types of orthoses	Moderate differences in temperature depending on orthosis type	JADAD: 2/PEDRO: 8	

**Table 4:** Summary of results.

Study	Sample	°C	SD
Jiménez-Pérez et al. [10]	30	2.5	1.0
Querol-Martinez et al. [18]	40	1.8	0.9
Gatt et al. [19]	50	3.0	1.2
Petrova et al. [21]	100	2.0	0.7
Villar Rodríguez et al. [22]	25	1.5	0.8
Lopes-Martins et al. [23]	30	2.2	1
